# Prevalence of Common Diseases in Indigenous People in Colombia

**DOI:** 10.3390/tropicalmed7060109

**Published:** 2022-06-18

**Authors:** Hannah Bauer, Gustavo Andrés Concha Mendoza, Lothar Kreienbrock, Maria Hartmann, Hagen Frickmann, Simone Kann

**Affiliations:** 1 Institute for Hygiene and Microbiology, Julius-Maximilians University, 97070 Würzburg, Germany; hannah.bauer2@stud-mail.uni-wuerzburg.de; 2 Organization Wiwa Yugumaiun Bunkanarrua Tayrona (OWYBT), Valledupar 200001, Colombia; gustavoconcha16@gmail.com; 3 Department of Biometry, Epidemiology and Information Processing, University of Veterinary Medicine Hannover, 30559 Hannover, Germany; lothar.kreienbrock@tiho-hannover.de (L.K.); maria.hartmann@tiho-hannover.de (M.H.); 4 Department of Microbiology and Hospital Hygiene, Bundeswehr Hospital Hamburg, 20359 Hamburg, Germany; frickmann@bnitm.de; 5 Institute for Medical Microbiology, Virology and Hygiene, University Medicine Rostock, 18057 Rostock, Germany; 6 Medical Mission Institute, 97074 Würzburg, Germany

**Keywords:** Chagas disease, indigenous, public health, Colombia, Sierra Nevada, neglected groups

## Abstract

The Indigenous tribe called the Wiwa lives retracted in the Sierra Nevada de Santa Marta, Colombia. Little is known about their health status and whether the health care system in place covers their needs. In 2017 and 2018, a permanent physician was in charge for the Wiwa. Diseases and complaints were registered, ranked, and classified with the ICD-10 coding. Datasets from the Indigenous health care provider Dusakawi, collected from local health points and health brigades travelling sporadically into the fields for short visits, were compared. Furthermore, a list of provided medication was evaluated regarding the recorded needs. The most common complaints found were respiratory, infectious and parasitic, and digestive diseases. The top ten diagnoses collected in the health points and in the health brigade datasets were similar, although with a different ranking. The available medication showed a basic coverage only, with a critical lack of treatment for many severe, chronic, and life-threatening diseases. Most of the detected diseases in the Indigenous population are avoidable by an improvement in health care access, an expansion of the provided medication, and an increase in knowledge, hygiene, and life standards.

## 1. Introduction

The Indigenous tribe called the Wiwa lives in the northeast of Colombia in the Sierra Nevada de Santa Marta. They settle in retracted areas and usually avoid contact to the outside world. Therefore, only little is known about their general health status, common diseases, and whether the accessible provided health care is sufficient or not. They live in poor conditions such as having no access to clean drinking water (unprotected wells, rivers), no electricity, no sanitation, and lacking basic infrastructure such as roads, etc. The Wiwas use traditional ways of farming (top dressing) and live close together with their livestock. In most cases, hospitals are far away (e.g., six to twelve hours walking distance) and health points are only sporadically occupied by a nurse and sparsely equipped with medication or medical tools, if in place at all.

According to the World Bank statistics (2020), 43% of the Indigenous people in the Americas live in poverty and about 24% in extreme poverty, which is more than twice the rate of the non-Indigenous population [1]. Only 0.47% of the health institutions within the General System of Social Security include Indigenous people, and 75% of these only offer first level services (e.g., low-complexity technology such as an X-ray, etc.) [2,3]. Furthermore, the Wiwas belong to a population at risk of extinction [2].

The Indigenous tribe has their own health care provider called Dusakawi, which is funded by the government. The main parts of the funds are used for preventive care measures such as vaccinations for children and dental care programs, which are performed by Dusakawi Health Brigades (Bs). These Bs visit certain Indigenous villages sporadically but repetitively for 1–2 days. As a medical doctor is part of the team, patients can contact them at that time. Other parts of the funds are used to employ nurses at several health points, who are occasionally available for short terms.

There are permanent and central health points, for example, in Valledupar, which is equipped with a doctor and a nurse. However, Valledupar is on average about six walking hours away from most Indigenous villages. Another problem for Indigenous people is that not all hospitals welcome them. Therefore, the Wiwas must consult certain governmental hospitals being in charge for them. Again, it is difficult for the Indigenous population to reach these hospitals, as some of them live even further away.

The aim of the study was (1) to find out the most common diseases that affect the indigenous communities; (2) to compare the gained data with the records collected by the health officials (health brigades and health points); and (3) to see whether any improvements in health care are needed and if so, in which way.

## 2. Materials and Methods

### 2.1. Ethical Approval

The study was performed according to the Declaration of Helsinki. The study was approved by the Ethics Committee of Santa Marta, Colombia (Acta No. 032018). Written informed consent was obtained from each participant or from the parent or legal guardian of a child prior to participation. All patients received their results and a treatment, if appropriate.

### 2.2. Study Design

#### 2.2.1. Collected Study Data (ST)

During the study “Program against Chagas Disease in the Indigenous Population of Colombia” (2017 and 2018), patients in four villages Tezhumake, Cherua, (Department Cesar), Ashintukwa, and Seminke, (Department La Guajira) were examined.

After the screening, Chagas positive tested patients were offered a drug observed treatment (DOT) for free. During the Chagas treatment phase of two months, a doctor was permanently in place to monitor the therapy. During that time, all village members—included into the DOT or not—could contact them at any time, if needed. For patients belonging to the DOT group, a study file was created; for those consulting the doctor who were not in the DOT group, the doctor created separate records. These DOT-independent records included information about the medical history (present complaints, general history, allergies, family history, general health status, etc.), age, sex, day of the consultation, results of the general physical examination and, if needed, further actions such as blood withdrawal, referrals, electrocardiography, and the resulting diagnoses. 

As there is no electricity in Wiwa villages, the study doctor as well as the doctors of the Dusakawi Health Points and Health Brigades first created a patient file in paper form. After their return to Valledupar, they entered the data into the database of the health care provider, Dusakawi (in form of Excel files), which were then transferred to our study database for further analysis.

Most Indigenous people in the region speak their own dialect, called Damana, and Spanish, however, some elderly people only speak Damana. In these cases, either our promotors, who were from the region and spoke both languages natively, or translators from the villages were in place. This was also the case for the Health Brigades and Health Points.

#### 2.2.2. Data from the Dusakawi Health Points (HP) and the Dusakawi Health Brigades (B)

Data from the HP in Valledupar, which is located at the campus of the Hospital Rosario Pumarejo de Lopes, was collected in 2017 and 2018. In addition, referrals to the emergency room and hospitalizations of Indigenous patients in the Hospital Rosario Pumarejo de Lopes in 2018 were included. Further data were obtained from health points located in the Indigenous area of interest (Becerril, Codazzi, La Paz, San Juan). Additionally, ICD-Codes from outpatients from 2017, who could not be administered to any health point, were also included.

The HP data comprised ICD-10 codes for the diagnoses or symptoms, sex, and age groups (<1 year old, 1–4 years old, 5–14 years old, 15–44 years old, 45–59 years old, and ≥60 years).

The Bs generally consist of a physician and an auxiliary nurse as well as an odontologist and an auxiliary dental nurse. Usually, the Bs only visit the villages for 1–2 days per month on an irregular basis, however, if in place, they also cover the patient’s needs. Exact plans on their service in the villages are not available. Given the shortness of time of their visits, they cannot conduct follow up assessments and so therapy is often symptom-related and the coding results appear in the category “unspecific”. The collected data included information on the diagnosis such as ICD-10 code, birthdate, date of examination, and sex.

After classifying all diagnoses and complaints by chapters of the ICD-10 system, the data were compared regarding these chapters and their frequencies, age groups, and sex.

As the health care provider data also included preventive measures and screenings (e.g., vaccinations, counselling for medical information such as reproductive measures, etc.), those associated codes were excluded from the analysis for the comparison with our data (ICD Chapter XXI: Z00-Z99).

To investigate whether the provided medications available in the field and in the health points cover the needs and are in line with the recommended standard therapies, research based on the guidelines and/or leading literature was performed. Second line treatments were also considered.

### 2.3. Data Management and Statistical Analyses

All data were coded and categorized following the International Statistical Classification of Diseases and Related Health Conditions (ICD-10) system of 2019 and subsequently ranked by frequency and classified by age and sex. Preventive measures and check-ups (e.g., vaccination, consultation for medical information such as reproductive measures, etc.) were excluded from the analysis (ICD chapter XXI: Z00-Z99).

All data were transferred into MS-Excel 2016^®^ spreadsheets and analyzed using SAS^®^, version 9.4 TS Level 1M7 (Cary, NC, USA) In detail, SAS was applied to stratify the Excel-dataset into categories in order to calculate the proportions for the descriptive analysis.

## 3. Results

### 3.1. Demographic Data

The physician collected the ST by consulting a total of 414 patients (194 (46.9%) males and 220 (53.1%) females) with one or more medical complaints, leading to a total number of 569 diagnoses and/or complaints (for further details, see Supplementary Table S1). The number of inhabitants during the study period was 775 in the four villages: 250 in Tezhumake, 155 in Seminke, 120 in Cherua, and 250 in Ashintukwa.

Overall, 121 people presented with more than one diagnosis. Out of those, 97 patients had more than one diagnosis, even during a single consultation. This led to an overall average number of diagnoses per person of 1.37 within a range from 1 to 5 (SD 0.67). The average age of the patients was 19.7 years (SD 18.0) with a median age of 13.5 years (minimum age 6 months, maximum age 86 years).

At the HPs, 154,415 diagnoses were registered. A total of 55,344 diagnoses were assigned to males and 99,071 to females. More detailed information on whether there were only one or more diagnoses per consultation was not available (for further details, see Supplementary Table S2).

The Bs collected 1810 diagnoses from 918 patients in 2017 and 2018 (for further details, see Supplementary Table S1). A total of 383 patients had more than one diagnosis and out of these, 46 patients had one or more diagnosis during a single consultation each. On average, each patient had 1.97 diagnoses within a range from 1 to 15 (SD 1.71) diagnoses. A total of the 436 (47.5%) patients were male, 482 (52.5%) were female. The average age was 19.4 years (SD 17.9) with a median age of 14 (minimum age one month, maximum age 86 years).

### 3.2. Diseases Diagnosed

In total, 569 diagnoses were registered within the ST by the study physician (Table 1). With 252 diagnoses (44.3%), “diseases of the respiratory system” (chapter X of ICD-10) were the most common diseases found. A total of 119 (20.9%) ICDs were assigned to chapter I “certain infectious and parasitic diseases”, followed by “diseases of the digestive system” (chapter XI) with 37 cases (6.5%), “diseases of the genitourinary system” (chapter XIV) with 33 consultations (5.8%), and “diagnoses of the skin and subcutaneous tissue” (chapter XII) with 30 patients (5.3%). The specific ranking and comparison of each village is summarized in Supplementary Table S3.

By stratifying the patient population into age groups as used by the health points (<1 year old, 1–4 years old, 5–14 years old, 15–44 years old, 45–59 years old, and ≥60), it can be concluded that “diseases of the respiratory system” (chapter X), “certain infectious and parasitic diseases” (chapter I), and “diseases of the ear and mastoid process” (chapter VIII), respectively, were more represented among children aged 0–14 years (59.9%, 65.5%, and 66.7%) than among adults older than 15 years (40.1%, 34.4%, 33.3%) In contrast, for “diseases of the digestive system” (chapter XI) and “diseases of the genitourinary system” (chapter XIV), the proportion of adults older than 15 years (91.9% and 87.9%) was much higher than the proportion of children (8.1%, 12.1%). Further details can be found in Table 1. Focusing on the sex-specific distribution, females had a higher proportion of “diseases of the genitourinary system” (chapter XIV) with 9.62% of the female study population. In contrast, in the male study population, this chapter was represented by just 1.17% of the patients.

Next to the main chapter classification, the data were further analyzed in subchapters of the ICD-10. Regarding these, the most common diseases belonging to respiratory infections (chapter X) were “acute upper respiratory infections” (subchapter J00-J06), which was the leading diagnose with 159 cases (27.9%), followed by 60 cases (10.6%) referring to “other acute lower respiratory infections” (subchapter J20-J22). A total of 59 ICDs (10.4%) comprised “intestinal infectious diseases” (subchapter A00-A09), 36 (6.3%) “helminthiases” (B65-B83), and 34 (6.0%) “diseases of the esophagus, stomach, and duodenum” (K20–K31). Further information about the subchapter classifications is summarized in Supplementary Table S4.

Within the HP collective, “diseases of the digestive system” (chapter XI) were most common with 36,381 cases (23.6%). In second position with 24,387 (15.8%) cases, “symptoms, signs, and abnormal clinical and laboratory findings, not elsewhere classified” (chapter XVIII) were recorded. In third place with 18,104 (11.7%) cases, “diseases of the respiratory system” (chapter X) were observed, followed by 14,123 (9.2%) diagnoses with “diseases of the genitourinary system” (chapter XIV) and 11.254 (7.3%) diagnoses with “certain infectious and parasitic diseases” (chapter I).

**Figure 1:**
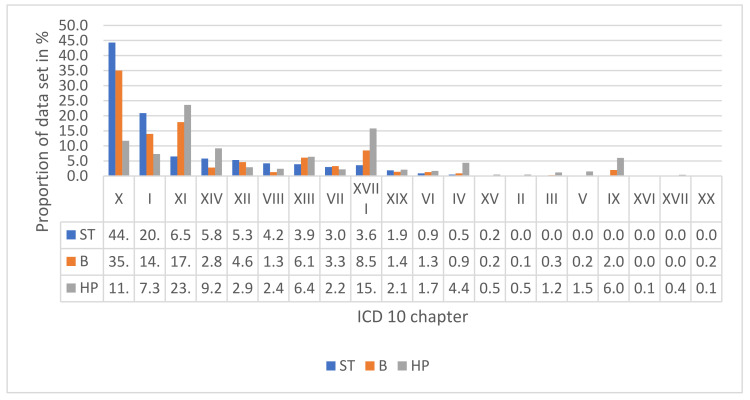
The comparison of the results of the three datasets, classified by the chapters of the ICD-10 2019: I = Certain infectious and parasitic diseases, II = Neoplasms, III = Diseases of the blood and blood-forming organs and certain disorders involving the immune mechanism, IV = Endocrine, nutritional, and metabolic diseases, V = Mental and behavioral disorders, VI = Diseases of the nervous system, VII = Diseases of the eye and adnexa, VIII = Diseases of the ear and mastoid process, IX = Diseases of the circulatory system, X = Diseases of the respiratory system, XI = Diseases of the digestive system, XII = Diseases of the skin and subcutaneous tissue, XIII = Diseases of the musculoskeletal system and connective tissue, XIV = Diseases of the genitourinary system, XV = Pregnancy, childbirth, and the puerperium, XVI = Certain conditions originating in the perinatal period, XVIII = Congenital malformations, deformations, and chromosomal abnormalities, XVIII = Symptoms, signs, and abnormal clinical and laboratory findings, not elsewhere classified, XIX = Injury, poisoning and certain other consequences of external causes, XX = External causes of morbidity and mortality. ST = Study data, B = Brigades, HP = Health points.

For the B collective in total, 1810 diagnoses were recorded. With 633 diagnoses (35.0%), “diseases of the respiratory system” (chapter X) were the most common diseases of the patients. A total of 324 (17.9%) ICDs were registered as “diseases of the digestive system” (chapter XI), followed by 254 (14.0%) “certain infectious and parasitic diseases” (chapter I), 154 (8.5%) “symptoms, signs and abnormal clinical and laboratory findings, not elsewhere classified” (chapter XVIII), and 111 (6.1%) “diseases of the musculoskeletal system and connective tissue” (chapter XIII). In total, the B and HP collective reported 24,541 cases assigned to chapter XVIII, which were not further classified. For the comparison of all three data sets, please see Table 2 and Figure 1.

The provided medication list (see Supplementary Table S5) was assessed regarding its coverage of the registered complaints and diseases in all three datasets. Tuberculosis and HIV were excluded from the list, because for those infections, specific centers are in place.

As a result, diseases were found that could be treated well, for example, mild forms of respiratory infections such as bronchitis, mild pneumonia without comorbidities, ascaridosis, and mild forms of hypertension.

Partially treatable (meaning some, but not all necessary medications were in place) were diseases such as bronchial asthma, amoebiasis, scabies, bacterial intestinal infections, type 2 diabetes, and gestational hypertension.

Examples for diseases that were not appropriately treatable at all were: moderate and severe pneumonia, hypertensive crisis, myocardial infarction, type I diabetes, hypoglycemia, epilepsy, anxiety, agitation, and postpartum hemorrhage, among others.

## 4. Discussion

The mortality and morbidity rates of Indigenous people in Latin America are higher compared to non-Indigenous people [1,2,4]. The Wiwa, an Indigenous tribe in the Sierra Nevada de Santa Marta, Colombia, lives in retracted areas and usually avoid contact with the outside world. Therefore, little is known about their most common diseases and their health status. Their access to health care and medical services is sparse. In addition, poor living conditions, no access to clean drinking water, missing sanitation, and low educational standards favor high rates of diseases [4,5].

During the study, 414 patients, comprising 55.6% of the Indigenous inhabitants, consulted the study physician once or several times during a two-month stay of the doctor. In total, 569 diagnoses/consults were registered (1.37 consults per person) in the collected study data (ST) and 1810 consultations in the data from the brigades (Bs) (1.97 consult per person). This clearly indicates a poor health status and a high need for medical care.

The collected ST indicated that respiratory tract diseases were the leading causes for the consultations (44.3%), followed by infectious and parasitic diseases (20.9%), and gastrointestinal problems (6.5%). This is in accordance with previously described findings [6], referring to the high mortality rates in vulnerable populations. Most causes of death among populations under poverty conditions worldwide are treatable gastrointestinal and respiratory diseases [7].

Comparing these results with the data registered by the Bs and at the HPs, the top 10 ST diagnoses were also found in their respective rankings, but in a slightly different order. Notably, in the data from the Bs, 8.5% (ranked 4) and 15.8% (ranked 2) in the HP data of the diagnoses were classified as “symptoms, signs, and abnormal clinical findings” (not present in the ST). These classifications demonstrate the lack of diagnostic possibilities, tools, and follow up measures for patients and medical staff, but also the need for permanent access to medical care.

Overall, the HP-derived data differed more from the ST than the B-derived data. It may be postulated that the health points need to be reached by foot and are therefore out of reach for severely ill patients. Conversely, the motivation to undertake the long journey to the health points with a mild illness will most likely not be sufficiently high. Presumably, this is why the ST data and B data may provide a more accurate picture of illnesses among the Indigenous people.

During the presence of the study doctor, many severe diagnoses were found (e.g., pneumonia, intestinal parasitosis, amoebiasis) that could be easily treated, potentially avoiding negative outcomes such as disabilities or even death, if the permanent availability of a physician could be guaranteed. Additionally, diseases occurred (e.g., puerperal fever) that needed to be treated in a hospital. This option, however, is often out of reach for Indigenous people, as infrastructure is locally missing.

Next to respiratory and infectious diseases, many cases of “diseases of the ear and mastoid process” and “diseases of the respiratory system” were reported in children younger than 14 years. This is in accordance with the findings described previously [8,9,10]. In contrast, “diseases of the digestive system” and “diseases of the genitourinary system” and “musculoskeletal system” occurred more frequently in patients above 15 years of age. Causatively, at about 12–15 years, the majority of Indigenous people are married and thus genitourinary diseases show up more frequently. Furthermore, the physically heavy field work favors musculoskeletal diseases, which become noticeable in older age groups.

In accordance with previously published data, genitourinary infections are more frequently found in females [11].

The main reasons for the quantitatively leading diseases, which are mainly preventable and avoidable, comprise the following: lack of access to clean drinking water (river, unprotected wells are used), insufficient hygiene management (e.g., no sanitation, living closely together with livestock), poor living conditions, poverty, missing knowledge/education as well as awareness, and as explained above, a lack of access to permanent medical care and sufficient medication [4,12]. These conditions are directly connected to higher mortality and morbidity rates [13,14].

In all of the assessed villages, the problems were comparable. However, in Cherua, the rate of respiratory infections was higher than in the other villages (52.6%), which can be explained by significant temperature drops at night (about 5 °C) due to the high altitude of this place in the mountains [15].

Notably, some ICD-10 chapters did not appear at all or only with very small quantity. Those rarely found diagnoses were: “neoplasms” (chapter II), “mental and behavioral disorders” (chapter V), “certain conditions originating in the perinatal period” (chapter XVI), and “congenital malformations, deformities, and chromosomal abnormalities” (chapter XVII). It is unlikely that these diseases do not appear in the communities, as they are described in the literature for Indigenous populations and also among the rest of the Colombian population and worldwide [16,17,18]. However, due to limited diagnostic and therapeutic tools (e.g., for neoplastic diseases), they are most likely neglected. In addition, psychiatric diseases are stigmatized and the awareness of mental health is low. Therefore, an underreporting is also likely.

The missing chapters “certain conditions originating in the perinatal period” (chapter XVI) and “congenital malformations, deformities and chromosomal abnormalities” (chapter XVII) were most likely not mentioned because many pregnancies do not reach the perinatal phase and instead lead to stillbirth (e.g., due to insufficient prenatal care). As reported by Roncancino, between 1999 and 2008, congenital anomalies were responsible for 3.4% of all fetal deaths and for 19.3% of all neonatal deaths in the country [19]. As peri- and postnatal care is also not available for Indigenous communities, congenital malformed or deformed children do not receive the needed attention and therefore die in most cases.

The analysis of the provided medication list showed that some mild to moderate diseases could be covered well (e.g., bronchitis). Other diseases are covered partially (e.g., diabetes I, hypertensive crisis, myocardial infarction, postpartum hemorrhage). If they clinically worsen (e.g., to status asthmaticus) or are associated with complications (e.g., hyper/hypoglycemia), patients cannot be treated appropriately. Furthermore, other severe diseases such as myocardial infarction cannot be treated at all. In particular, the missing medication during pregnancy and birth can be blamed as a cause of the high maternal and child mortality rates among the Indigenous population in Colombia [2].

The problem of poor health care associated with higher morbidity among the Indigenous populations is not limited to the inhabitants of the Sierra Nevada de Santa Marta. This can also be observed in other countries with regard to the number of communicable diseases [20,21,22,23,24], non-communicable diseases [25,26], or oral health [27]. Unfortunately, datasets on many indigenous people in other countries are incomplete or missing completely. An assessment from 2013 showed that with regard to sexually transmitted diseases, there are gaps in the data on the infection rates among indigenous people worldwide. The available data, however, still indicate a higher infection rate among Indigenous compared to non-Indigenous people [20,21]. This can be observed not only in resource-poor countries, but also in resource-rich industrialized countries such as Australia [21,22].

As limitations of the study, it needs to be mentioned that the HP data provided did not allow for any precise conclusions on the number of persons claiming more than one complaint, but only on the number of diagnoses. As above-mentioned, we had to use the age groups given as we did not obtain the specific age of each patient. In addition, it would have been good if the study doctor could have stayed longer in the villages, so that more data could have been collected. However, as the study duration was limited, a longer stay was not possible.

## 5. Conclusions

The most common diseases registered in the Indigenous population were respiratory, infectious, and parasitic as well as digestive diseases. The data demonstrate an essential need for sustainable medical access, a necessity for infrastructure improvements, and a demand for an upgrade of the available medication and diagnostics. Accordingly, future research should focus on the most appropriate ways of implementing diagnostic and therapeutic strategies in order to better address the identified medical needs of the Indigenous people. Such implementation research should focus on the availability, affordability, and sustainability of the assessed strategies, because all three needs must be met to allow for beneficial long-term effects on the health of Indigenous populations.

## Figures and Tables

**Table 1:** The ranking, number, and percentage of all of the recorded diagnoses in the collected study data (ST), assigned to chapters of the International Statistical Classifications of Diseases and Related Health Conditions (ICD-10) of 2019.

Ranking.	ICD Chapter	Total	Age in Years No. of Diagnoses (%) *					
			<1	1–4	5–14	15–44	45–59	≥60
1	X Diseases of the respiratory system	252 (44.3)	8 (53.3)	49 (49.5)	94 (54.7)	76 (35.8)	16 (36.4)	9 (33.3)
2	I Certain infectious and parasitic diseases	119 (20.9)	3 (0.2)	32 (32.3)	43 (25.0)	32 (15.1)	6 (13.6)	3 (11.1)
3	XI Diseases of the digestive system	37 (6.5)	0 (0.0)	1 (1.0)	2 (1.2)	30 (14.2)	2 (4.5)	2 (7.4)
4	XIV Diseases of the genitourinary system	33 (5.8)	0 (0.0)	2 (2.0)	2 (1.2)	22 (10.4)	6 (13.6)	1 (3.7)
5	XII Diseases of the skin and subcutaneous tissue	30 (5.3)	1 (0.7)	7 (7.1)	8 (4.7)	12 (5.7)	1 (2.3)	1 (3.7)
6	VIII Diseases of the ear and mastoid process	24 (4.2)	1 (0.7)	4 (4.0)	11 (6.4)	4 (1.9)	2 (4.5)	2 (7.4)
7	XIII Diseases of the musculoskeletal system and connective tissue	22 (3.9)	0 (0.0)	0 (0.0)	0 (0.0)	14 (6.7)	7 (15.9)	1 (3.7)
8	VII Diseases of the eye and adnexa	17 (2.9)	0 (0.0)	1 (1.0)	3 (1.7)	8 (3.8)	1 (2.3)	4 (14.8)
9	XVIII Symptoms, signs and abnormal clinical and laboratory findings, not elsewhere classified	15 (2.6)	1 (0.7)	0 (0.0)	1 (0.6)	9 (4.2)	1 (2.3)	3 (11.1)
10	XIX Injury, poisonings and certain other consequences of external causes	11 (1.9)	1 (0.7)	1 (1.0)	5 (2.9)	3 (1.4)	1 (2.3)	0 (0.0)
11	VI Diseases of the nervous system	5 (0.9)	0 (0.0)	0 (0.0)	3 (1.7)	0 (0.0)	1 (2.3)	1 (3.7)
12	IV Endocrine, nutritional and metabolic diseases	3 (0.5)	0 (0.0)	2 (2.0)	0 (0.0)	1 (0.5)	0 (0.0)	0 (0.0)
13	XV Pregnancy, childbirth and the puerperium	1 (0.2)	0 (0.0)	0 (0.0)	0 (0.0)	1 (0.5)	0 (0.0)	0 (0.0)
total		569	15	99	172	212	44	27

* Percentages refer to the total number of diagnoses in a column.

**Table 2:** The comparison of the ICD chapters for all datasets. ST = Study data, H = health points, B = brigades.

	ST		HP		B	
ICD-10 Chapter *	Ranking	No. of Diagnoses (%)	Ranking	No. of Diagnoses (%)	Ranking	No. of Diagnoses (%)
X	1	252 (44.3)	3	18,104 (11.7)	1	633 (35.0)
I	2	119 (20.9)	5	11,254 (7.3)	3	254 (14.0)
XI	3	37 (6.5)	1	36,381 (23.6)	2	324 (17.9)
XIV	4	33 (5.8)	4	14,123 (9.2)	8	50 (2.8)
XII	5	30 (5.3)	9	6042 (3.9)	6	83 (4.6)
VIII	6	24 (4.2)	14	2144 (1.4)	12	23 (1.3)
XIII	7	22 (3.9)	6	9845 (6.4)	5	111 (6.1)
VII	8	17 (3.0)	10	3462 (2.2)	7	59 (3.3)
XVIII	9	15 (2.6)	2	24,387 (15.8)	4	154 (8.5)
XIX	10	11 (1.9)	11	3239 (2.1)	10	25 (1.4)
VI	11	5 (0.9)	12	2567 (1.7)	11	24 (1.3)
IV	12	3 (0.5)	8	6815 (4.4)	13	16 (0.9)
XV	13	1 (0.2)	16	736 (0.5)	16	3 (0.2)
IX		0 (0.0)	7	9329 (6.0)	9	37 (2.0)
III		0 (0.0)	15	1906 (1.2)	14	6 (0.3)
XX		0 (0.0)	19	184 (0.1)	15	4 (0.2)
V		0 (0.0)	13	2385 (1.5)	16	3 (0.2)
II		0 (0.0)	17	702 (0.5)	18	1 (0.1)
XVI		0 (0.0)	20	161 (0.1)	-	0 (0.0)
XVII		0 (0.0)	18	658 (0.4)	-	0 (0.0)
Total		569		154,415		1810

* I = Certain infectious and parasitic diseases, II = Neoplasms, III = Diseases of the blood and blood-forming organs and certain disorders involving the immune mechanism, IV = Endocrine, nutritional and metabolic diseases, V = Mental and behavioral disorders, VI = Diseases of the nervous system, VII = Diseases of the eye and adnexa, VIII = Diseases of the ear and mastoid process, IX = Diseases of the circulatory system, X = Diseases of the respiratory system, XI = Diseases of the digestive system, XII = Diseases of the skin and subcutaneous tissue, XIII = Diseases of the musculoskeletal system and connective tissue, XIV = Diseases of the genitourinary system, XV = Pregnancy, childbirth and the puerperium, XVI = Certain conditions originating in the perinatal period, XVII = Congenital malformations, deformations, and chromosomal abnormalities, XVIII = Symptoms, signs, and abnormal clinical and laboratory findings, not elsewhere classified, XIX = Injury, poisoning, and certain other consequences of external causes, XX = External causes of morbidity and mortality.

## Data Availability

All relevant data are presented in the manuscript and in the Supplementary Materials. Raw data can be made available at reasonable request.

## Supplementary Materials

The following supporting information can be downloaded at: https://www.mdpi.com/article/10.3390/tropicalmed7060109/s1, Table S1: The number of patients and detected diagnoses in all four Wiwa villages in the collected study data (ST) and the data provided by the brigades (B); Table S2: Diagnoses from the health posts (HP) between 2017 and 2018; Table S3: The overall comparison of the ICD chapters in the collected study data (ST) for each village; Table S4: The ranking of number and percentage of all found diagnoses assigned to subchapters of the International Statistical Classification of Diseases and Related Health Conditions (ICD 10) of 2019; Table S5: The medication list provided by Dusakawi.
