# Supplementary material for: Prevalence of Common Diseases in Indigenous People in Colombia
Source: Trop Med Infect Dis. 2022 Jun 18;7(6):109. doi: 10.3390/tropicalmed7060109 (PMC9231329; doi:10.3390/tropicalmed7060109)
Supplement: Supplementary file 1 [file tropicalmed-07-00109-s001.zip › tropicalmed-1726014-supplementary.pdf]

Tropical Medicine and Infectious Disease

MDPI

Supplementary Materials: Prevalence of Common Diseases in Indigenous People in Colombia

Hannah Bauer [1], Gustavo Andrés Concha Mendoza [2], Lothar Kreienbrock [3], Maria Hartmann [3], Hagen Frickmann [4,5] and Simone Kann [6,*]

Table S1. The number of patients and detected diagnoses in all four Wiwa villages in the collected study data (ST) and the data provided by the brigades (B).

Village[1]	ST						B					
	No. Patients (%)			No. Diagnoses (%)			No. Patients (%)			No. Diagnoses (%)		
	f[2]	m[3]	Total	f	m	Total	f	m	Total	f	m	Total
T	110 (55.6)	88 (44.4)	198	165 (55.9)	130 (44.1)	295	120 (50.8)	116 (49.2)	236	177 (55.1)	144 (44.9)	321
S	53 (49.1)	55 (50.9)	108	70 (51.5)	66 (48.5)	136	58 (59.2)	40 (40.8)	98	58 (59.2)	40 (40.8)	98
C	31 (56.4)	24 (43.6)	55	45 (57.7)	33 (42.3)	78	156 (58.2)	112 (41.8)	268	305 (59.0)	212 (41.0)	517
A	26 (49.1)	27 (50.9)	53	32 (53.3)	28 (46.7)	60	158 (47.6)	174 (52.4)	332	508 (58.1)	366 (41.9)	874
Total 2	220 (53.1)	194 (46.9)	414	312 (54.8)	257 (45.2)	569	482 (52.5)	436 (47.5)	918	1048 (57.9)	762 (42.1)	1810

[1] T = Tezhumake, S = Seminke, C = Cherua, A = Ashintukwa. [2] Female. [3] Male.

Table S2. Diagnoses from the health posts (HP) between 2017 and 2018.

HP	Diagnoses				
	f[1]		m[2]		
	n	%	n	%	Total
Valledupar	50,592	65.1	27,149	34.9	77,741
San Juan	12,604	64.7	6879	35.3	19,483
Codazzi	11,416	62.0	6988	38.0	18,404
Additional Wiwas	8671	64.9	4684	35.1	13,355
ER[3] Valledupar	5822	63.5	3344	36.5	9166
Becerrill	5191	62.2	3151	37.8	8342
La Paz	4112	64.2	2296	35.8	6408
Hospitalization	663	43.7	853	56.3	1516
Total	99,071	64.2	55,344	35.8	154,415

[1] Male. [2] Female. [3] Emergency room.

Table S3. The overall comparison of the ICD chapters in the collected study data (ST) for each village.

No. of ICD Chapter *	T		S		C		A		Overall Ranking
	No. of Diseases (% of Population)	Ranking	No. of Diseases (% of Population))	Ranking	No. of Diseases (% of Population))	Ranking	No. of Diseases (% of Population))	Ranking	
X	138 (55.2)	1	53 (34.2)	1	41 (34.2)	1	20 (8.0)	1	1
I	49 (19.6)	2	43 (27.7)	2	10 (8.3)	2	17 (6.8)	2	2
XI	19 (7.6)	4	11 (7.1)	3	4 (3.3)	5	3 (1.2)	4	3
XIV	20 (8.0)	3	6 (3.9)	4	4 (3.3)	5	3 (1.2)	4	4
XII	15 (6.0)	5	5 (3.2)	5	8 (6.7)	3	2 (0.8)	5	5
VIII	14 (5.6)	6	4 (2.6)	6	2 (1.7)	6	4 (1.6)	3	6

XIII	9 (3.6)	7	5 (3.2)	5	5 (4.2)	4	3 (1.2)	4	7
XVIII	15 (6.0)	5							9
VII	6 (2.4)	8	5 (3.2)	5	2 (1.7)	6	4 (1.6)	3	8
XIX	5 (2.0)	9	3 (1.9)	7	1 (0.8)	7	2 (0.8)	5	10
VI	2 (0.8)	10	1 (0.6)	8	1 (0.8)	7	1 (0.4)	6	11
IV	2 (0.8)	10					1 (0.4)	6	12
XV	1 (0.4)	11							13
All diseases	295		136		78		60		569
All patients	198		108		55		53		414
All inhabitants	250		155		120		250		775

* I = Certain infectious and parasitic diseases, IV = Endocrine, nutritional and metabolic diseases, VI = Diseases of the nervous system, VII = Diseases of the eye and adnexa, VIII = Diseases of the ear and mastoid process, X = Diseases of the respiratory system, XI = Diseases of the digestive system, XII = Diseases of the skin and subcutaneous tissue, XIII = Diseases of the musculoskeletal system and connective tissue, XIV = Diseases of the genitourinary system, XV = Pregnancy, childbirth and the puerperium, XVIII = Symptoms, signs, and abnormal clinical and laboratory findings, not elsewhere classified, XIX = Injury, poisoning, and certain other consequences of external causes.

Table S4. The ranking of number and percentage of all found diagnoses assigned to subchapters of the International Statistical Classification of Diseases and Related Health Conditions (ICD 10) of 2019.

Number or ICD 10 Subchapter	ICD 10 Subchapter	Number of Diagnoses	Percentage (%)	Ranking
J00-J06	Acute upper respiratory infections	159	27.9	1
J20-J22	Other acute lower respiratory infections	60	10.6	2
A00-A09	Intestinal infectious diseases	59	10.4	3
B65-B83	Helminthiases	36	6.3	4
K20-K31	Diseases of esophagus, stomach and duodenum	34	6.0	5
M40-M54	Dorsopathies	21	3.7	6
N30-N39	Other diseases of urinary system	19	3.3	7
H65-H75	Diseases of middle ear and mastoid	16	2.8	8
B35-B49	Mycoses	16	2.8	8
J09-J18	Influenza and pneumonia	16	2.8	8
L00-L08	Infections of the skin and subcutaneous tissue	13	2.3	9
R50-R69	General symptoms and signs	12	2.1	10
H10-H13	Disorders of Conjunctiva	10	1.8	11
J30-J39	Other diseases of upper respiratory tract	10	1.8	11
L40-L45	Papulosquamous disorders	9	1.6	12
N70-N77	Inflammatory diseases of female pelvic organs	6	1.1	13
H15-H22	Disorders of sclera, cornea, iris and ciliary body	6	1.1	13
L20-L30	Dermatitis and eczema	6	1.1	13
B85-B89	Pediculosis, ascariasis and other infestations	5	0.9	14
J40-J47	Chronic lower respiratory diseases	5	0.9	14
H60-H62	Diseases of external ear	5	0.9	14
N80-N98	Noninflammatory disorders of female genital tract	4	0.7	15
H80-H83	Diseases of inner ear	3	0.5	16
T20-T32	Burns and corrosions	3	0.5	16
K00-K14	Diseases of oral cavity, salivary glands, and jaws	2	0.4	17
E40-E46	Malnutrition	2	0.4	17
B00-B09	Viral infections characterized by skin and mucous membrane lesions	2	0.4	17
J95-J99	Other diseases of the respiratory system	2	0.4	17
N20-N23	Urolithiasis	2	0.4	17
G50-G59	Nerve, nerve root and plexus disorders	2	0.4	17
S90-S99	Injuries to the ankle and foot	2	0.4	17

L80-L99	Other disorders of the skin and subcutaneous tissue	2	0.4	17
N10-N19	Renal tubulo-interstitial diseases	1	0.2	18
O85-O92	Complications predominantly related to the puerperium	1	0.2	18
M80-M94	Osteopathies and chondropathies	1	0.2	18
S50-S59	Injuries to the elbow and forearm	1	0.2	18
R00-R09	Symptoms and signs involving the circulatory and respiratory system	1	0.2	18
R10-R19	Symptoms and signs involving the digestive system and abdomen	1	0.2	18
B99-B99	Other infectious diseases	1	0.2	18
K80-K87	Disorders of the gallbladder, biliary tract, and pancreas	1	0.2	18
N60-N64	Disorders of the breast	1	0.2	18
T08-T14	Injuries to unspecified part of trunk, limb or body region	1	0.2	18
G80-G83	Cerebral palsy and other paralytic syndromes	1	0.2	18
T51-T65	Toxic effects of substances chiefly nonmedicinal as source	1	0.2	18
G40-G47	Episodic and paroxysmal disorders	1	0.2	18
T15-T19	Effects of foreign body entering through natural orifice	1	0.2	18
G20-G26	Extrapyramidal and movement disorders	1	0.2	18
H00-H06	Disorders of eyelid, lacrimal system and orbit	1	0.2	18
S60-S69	Injuries to the wrist and hand	1	0.2	18
R30-R39	Symptoms and signs involving the urinary system	1	0.2	18
S20-S29	Injuries to the thorax	1	0.2	18
E65-E68	Obesity and other hyperalimentation	1	0.2	18
Total		569		

Table S5. The medication list provided by Dusakawi.

Substance	Dose	Pharmaceutical Form
Acetylsalicylic acid	100 mg	Tablet
Acyclovir	200 mg	Tablet
	5% 15 g	Cream
Adrenaline	1 mg	Vial
Albendazole	100 mg/5 mL 20 mL	Suspension
	200 mg	Tablet
Allopurinol	100 mg,	Tablet
	300 mg	Tablet
Alpha methyldopa	250 mg	Tablet
Aluminum hydroxide + magnesium + Simeticone	360 mL	Suspension
Amantadine	100 mg	Capsule
Amikacin	100 mg	Vial
	500 mg	Vial
Aminophylline	240 mg	Vial
Amiodarone	150 mg	Vial
	200 mg	Tablet
Amitriptyline	25 mg	Tablet
Amlodipine	5 mg	Tablet
	10 mg	Tablet
Amoxicillin	250 mg/100 mL	Suspension
	500 mg	Capsule
	500 mg/100 mL	Suspension
Ampicillin	500 mg	Vial
	1 g	Vial

	250 mg x 60 mL	Suspension
	500 mg	Capsule
Anatoxina tetanica	0.5 mL	Vial
Ascorbic acid	100 mg/mL 30 mL	Oral drops
	500 mg	Chewable tablet
Atorvastatin	20 mg, 40 mg	Tablet
Atropine (sulfate)	1 mg	Vial
Azithromycin	200 mg x 15 mL	Suspension
	500 mg	Tablet
Beclomethasone	50 mcg	Nasal inhalator
	250 mcg	Oral inhalator
Benzyl benzoate	30%	Lotion
Betamethasone	0.05%	Cream
	3 mg	Vial
	4 mg	Vial
	8 mg	Vial
Betamethasone + Clotrimazole + Neomycin	0.04% + 1% + 0.5 g	Cream
Beta-methyldigoxin	0.1 mg	Tablet
	0.6 mg	Oral drops
	2 mL	Vial
Biperiden	2 mg	Tablet
Brimonidine (tartrate)	5 mL	Eye drops
Butylscopalamine bromide (Hioszinbutyl bromide)	10 mg	Tablet
Calcium + Vitamin D	600 + 200 IU	Tablet
Captopril	25 mg	Tablet
	50 mg	Tablet
Carbamazepine	2%	Oral suspension
	200 mg	Tablet
Carvedilol	6.25 mg	Tablet
	12.5 mg	Tablet
	25 mg	Tablet
Cefalexin	500 mg	Capsule
	250 mg/5mL	Suspension
Cefradine	500 mg	Tablet
	1 g	Vial
Ceftriaxone	1 g	Vial
Chloroquine	250 mg	Tablet
Chlorphenamine	2 mg/5 mL 120 mL	Syrup
	4 mg	Tablet
Ciprofloxacin	500 mg	Tablet
Clarithromycin	250 mg	Suspension
	500 mg	Tablet
Clonidine chlorohydrate	150 mg	Tablet
Clopidogrel	75 mg	Tablet
Clotrimazole	1%	Topical cream
	1%	Vaginal cream
	100 mg	Vaginal tablet
	30 mL	Lotion
Colchicine	0.5 mg	Tablet
Colistin + Neomycin		Ear drops
Conjugated estrogens	0.625 mg	Tablet
Cotrimocazole (Trimethoprim + Sulfmeth-oxazole)	160 mg + 800 mg	Tablet
	40 mg + 200 mg/5 mL 120 mL	Suspension
Cromoglicate	4%	Nose drops
	4%	Eye drops
Crotamiton	10% x 60 mL	Lotion

Dexamethasone	4 mg	Vial
	8 mg	Vial
Diclofenac	50 mg	Tablet
	75 mg	Vial
Dicloxacillin	250 mg x 80 mL	Suspension
	500 mg	Capsule
Dihydrocodeine	2.42 120 mL	Syrup
Dimenhydrinate	50 mg	Tablet
	50 mg	Capsule
Diphenhydramine	12.5 mg/5 mL 120 ml	Syrup
	100 mg	Tablet
Doxycycline	100 mg	Tablet
Enalapril	5 mg	Tablet
	20 mg	Tablet
Ergotamine + Caffeine	1 mg + 100 mg	Tablet
Erythromycin	500 mg	Tablet
	250 mg	Suspension
Esomeprazole	20 mg	Tablet
	40 mg	
Fluconazole	200 mg	Tablet
	50 mg/5 mL 20 ml	Suspension
Fluoxetine	20 mg	Tablet
Folic acid	1 mg	Tablet
Furosemide	40 mg	Tablet
Gemfibrozil	600 mg	Tablet
Gentamicin	0.3%	Eye drops
	80 mg	Vial
Glibenclamide	5 mg	Tablet
Haloperidol	5 mg	Tablet
Hartmann solution	500 mL	Solution for injection
Hydrochlorothiazide	25 mg	Tablet
Hydrocortisone	0.5%	Lotion
	1%	Topical cream
	100 mg	Vial
Hydrocortisone + Benzocaine		Rectal cream
Hydroxyzine	100 mg	Vial
Ibuprofen	400 mg	Tablet
Ipratropium bromide		Inhalator
Iron fumarate + Folic acid + Ascorbic acid (vitamin c)		Tablet
Iron sulfate	125 mg/mL 20 mL	Oral drops
	300 mg	Tablet
	600 mg/15 mL 120 mL	Syrup
Isosorbide	5 mg	Sublingual tablet
Isosorbide dinitrate	10 mg	Tablet
Ketoconazole	100 mg 60 mL	Suspension
	200 mg	Tablet
Ketotifen	1 mg	Tablet
	1 mg/5 mL x 100 mL	Syrup
Levodopa + Carbidopa	250 mg + 25 mg	Tablet
Levomepromazine	25 mg	Tablet
	100 mg	Tablet
	4%	Oral drops
Levonorgestrel	0.03 mg	Tablet
Levonorgestrel + Ethinylestradiol	100 mcg + 20 mcg	Tablet
Levothyroxine	25 mcg	Tablet
	50 mcg	Tablet

	100 mcg	Tablet
	125 mcg	Tablet
	150 mcg	Tablet
Lidocaine chlorohydrate	2%	Gel
Loratadine	5 mg/5 mL x 100 mL	Syrup
	10 mg	Tablet
Losartan	50 mg	Tablet
	100 mg	Tablet
Losartan + Hydrochlorothiazide	100 mg + 25 mg	Tablet
Mebendazole	100 mg	Suspension
	100 mg	Tablet
Medroxyprogesterone + Estradiol	25 mg + 5 mg	Vial
Medroxyprogesterone acetate	50 mg/mL (5%)	Vial
Metamizole	1g/2 mL	Vial
	2.5 g	Vial
Metformin	850 mg	Tablet
Methocarbamol	750 mg	Tablet
Methotrexate	2.5 mg	Tablet
Methylprednisolone	500 mg	Vial
Metoclopramide	10 mg	Tablet
	10 mg	Vial
Metoprolol succinate	25 mg	Tablet
	50 mg	Tablet
	100 mg	Tablet
Metoprolol tartrate	50 mg	Tablet
	100 mg	Tablet
Metronidazole	250 mg/5 mL	Suspension
	500 mg	Ovule
	500 mg	Tablet
Nalidixic acid	250 mg/120 mL	Suspension
Naproxen	2.5% 80 mL	Suspension
Naproxen + Caffeine	220 mg + 50 mg	Tablet
Neomycin + Polymyxin + Dexamethasone		Eye drops
Nifedipine	10 mg	Capsule
	30 mg	Tablet
Nimodipine	30 mg	Tablet
Nitrofurantoin	100 mg	Capsules
Nitrofurazone	500 mg	Topical cream
Norfloxacin	400 mg	Tablet
Nystatin	100,000 IU	Suspension
	100,000 IU	Vaginal tablet
	500,000 IU	Tablet
Nystatin + Zinc oxide		Topical cream
Olanzapine	5 mg	Tablet
	10 mg	Tablet
Omeprazole	20 mg	Capsule
Oxacillin	1 g	Vial
Oxymetazoline	0.25%, 0.05%	Nasal drops
Paracetamol	100 mg/mL x 30 mL	Oral drops
	150 mg/mL x 90 mL	Syrup
	500 mg	Tablet
Penicillin	1,000,000 IU	Vial
	1,200,000 IU	Vial
	2,400,000 IU	Vial
Phenytoin	100 mg	Tablet
	250 mg/5 mL	Vial
Physiological serum	500 mL	Bag

		Nasal drops
Pipotiazine	25 mg/1 mL	Vial
Potassium chloride	10 mL	Vial
Prednisolone	5 mg	Tablet
Propranolol	40 mg	Tablet
	80 mg	Tablet
Pyrantel pamoate	250 mg	Tablet
	250 mg/5 mL x 15 mL	Suspension
Ranitidine	300 mg	Tablet
	50 mg	Vial
Risperidone	1 mg	Tablet
Salbutamol	100 mcg	Inhalator
		Salt to nebulize
	2 mg/5 mL 120 mL	Syrup
Salts for oral rehydration		Bag
Silver sulfadiazine	1%	Cream
Sodium chloride	10 mL	Vial
Spironolactone	25 mg	Tablet
	100 mg	Tablet
Sucralfate	1 g	Tablet
Theophylline	125 mg	Tablet
	80 mg/15 mL	Elixir
Timolol maleate	0.5%	Eye drops
Tinidazole	500 mg	Tablet
Tramadol	100 mg/10 mL	Oral drops
	100 mg/mL	Vial
Tranexamic acid	500 mg	Tablet
Trazodone	50 mg	Tablet
Trimebutine	200 mg	Tablet
Valproate	250 mg	Soft capsule
	250 mg/5 mL 120 mL	Syrup
Valsartan	80 mg	Tablet
	160 mg	Tablet
Valsartan + Hydrochlorothiazide	160 mg + 12.5 mg	Tablet
	80 mg + 12.5 mg	Tablet
Vecuronium bromide	10 mg	Vial
Verapamil	80 mg	Tablet
	120 mg	Tablet
	240 mg	Tablet
Vitamin A (Retinol)	50,000 IU	Soft capsule
Vitamin B1 (Thiamine)	10 mL	Vial
	300 mg	Tablet
Vitamin B12	2 mL	Vial
Vitamin K	10 mg	Vial
Warfarin sodica	5 mg	Tablet
Zinc sulfate	2 mg/5 mL 120 mL	Syrup
